# The effects of a tourniquet used in total knee arthroplasty: a meta-analysis

**DOI:** 10.1186/1749-799X-9-13

**Published:** 2014-03-06

**Authors:** Wei Zhang, Ning Li, Sifeng Chen, Yang Tan, Mohammed Al-Aidaros, Liaobin Chen

**Affiliations:** 1 Department of Orthopedics, Zhongnan Hospital of Wuhan University, Wuhan, Hubei 430071, China; 2 Department of Orthopedics, The First Affiliated Hospital of Zhengzhou University, Zhengzhou 450002, China

**Keywords:** Tourniquet, Total knee arthroplasty, Blood loss, Complications

## Abstract

**Background:**

The purpose of this research is to evaluate the effects of a tourniquet in total knee arthroplasty (TKA).

**Methods:**

The study was done by randomized controlled trials (RCTs) on the effects of a tourniquet in TKA. All related articles which were published up to June 2013 from Medline, Embase, and Cochrane Central Register of Controlled Trails were identified. The methodological quality of the included studies was assessed by the Physiotherapy Evidence Database (PEDro) scale. The meta-analysis was performed using Cochrane RevMan software version 5.1.

**Results:**

Thirteen RCTs that involved a total of 689 patients with 689 knees were included in the meta-analysis, which were divided into two groups. The tourniquet group included 351 knees and the non-tourniquet group included 338 knees. The meta-analysis showed that using a tourniquet in TKA could reduce intraoperative blood loss (weighted mean difference (WMD), -198.21; 95% confidence interval (CI), -279.82 to -116.60; *P* < 0.01) but did not decrease the calculated blood loss (*P* = 0.80), which indicates the actual blood loss. Although TKA with a tourniquet could save the operation time for 4.57 min compared to TKA without a tourniquet (WMD, -4.57; 95% CI, -7.59 to -1.56; *P* < 0.01), it had no clinical significance. Meanwhile, the use of tourniquet could not reduce the possibility of blood transfusion (*P* > 0.05). Postoperative knee range of motion (ROM) in tourniquet group was 10.41° less than that in the non-tourniquet group in early stage (≤10 days after surgery) (WMD, -10.41; 95% CI, -16.41 to -4.41; *P* < 0.01). Moreover, the use of a tourniquet increased the risk of either thrombotic events (risk ratio (RR), 5.00; 95% CI, 1.31 to 19.10; *P* = 0.02) or non-thrombotic complications (RR, 2.03; 95% CI, 1.12 to 3.67; *P* = 0.02).

**Conclusions:**

TKA without a tourniquet was superior to TKA with a tourniquet in thromboembolic events and the other related complications. There were no significant differences between the two groups in the actual blood loss. TKA with a tourniquet might hinder patients' early postoperative rehabilitation exercises.

## Background

Total knee arthroplasty (TKA) is commonly performed using a tourniquet. A recent survey reported that 95% of the members of the American Association of Hip and Knee surgeons used a tourniquet for TKA [1]. Most orthopedic surgeons believed that extensive soft tissue release and bone cuts could result in higher blood loss in TKA. However, the application of a tourniquet offered better visualization of the structures and reduced intraoperative blood loss, which theoretically would facilitate the cementing quality and other surgical procedures [2]. Furthermore, the tourniquet could help reduce the operation time. Yavarikia et al. reported that the use of a tourniquet in TKA resulted in a much shorter operation time [3]. A prospective study by Willis-Owen et al. included 3,449 total knee arthroplasty showed that a prolonged operating time was associated with an increased incidence of infection [4].

However, some studies reported that several complications were associated with the use of a tourniquet in TKA, including skin blistering, wound hematoma, wound ooze, muscle injury, rhabdomyolysis, nerve palsy, postoperative stiffness, deep vein thrombosis (DVT), and pulmonary embolism (PE) [3-10]. Additionally, complications might interfere with the postoperative functional recovery and could result in unnecessary discomfort for the patients. There were some reports of severe and fatal complications following TKA with a tourniquet, which included pulmonary edema [11], renal failure [10], and PE that led to death in some cases.

Several randomized controlled trials (RCTs) comparing the effects of tourniquet use or not in TKA have been published, but these studies had different endpoints. Specially, TKA has become a standard operative procedure for relieving pain and restoring function [12], and there is no published meta-analysis comparing the use of a tourniquet and summarized statistical comparison of postoperative function recovery. Therefore, it is necessary to have a latest meta-analysis to summarize those issues. This study reviews the effects of a tourniquet use in TKA in order to provide an evidence for clear clinical guidance.

## Methods

The following criteria were required for inclusion: (1) patients underwent unilateral primary TKA, (2) a RCT comparing patients undergoing TKA with or without a tourniquet, (3) patients suffering from primary osteoarthritis (OA) or rheumatoid arthritis (RA), and (4) full text must be published in English.

The following criteria were required for exclusion: (1) bilateral TKA, revision TKA, and complicated TKA; (2) animal studies.

Electronic databases (Medline, Embase, and Cochrane Central Register of Controlled Trails) were searched by two independent researchers (ZW and CSF), which were published up to June 2013. The key words used in the search were ‘total knee arthroplasty’ or ‘total knee replacement’ and ‘tourniquet’ and ‘randomized controlled trial’. Reference lists of the relevant papers were also looked through for any further relevant studies.

The methodological quality of each included RCT was assessed by two independent researchers (ZW and CSF) by the Physiotherapy Evidence Database (PEDro) scale [13]. Because the PEDro score demonstrated moderate inter-rater reliability [intraclass correlation coefficient = 0.68; (95% CI: 0.57–0.76)] for clinical trials [14]. A trial with a score of 6 or more was considered high quality. All disagreements were resolved by the corresponding author (CLB).

Two authors (ZW and CSF) extracted relevant data, including sample size, study design, patient age, sex, body mass index (BMI), operation time, the intraoperative blood loss, overt blood loss, calculated blood loss, number of transfusion, knee range of motion (ROM), thromboprophylaxis, drainage system, thromboembolic events, and the non-thrombotic complications.

The meta-analysis was conducted with Cochrane Collaboration Review Manager 5.1 software. For continuous data, a weighted mean difference (WMD) and 95% confidence interval (CI) was used. For dichotomous outcomes, risk ratio/relative risk (RR) and 95% CI were calculated as the summary statistics. The statistical heterogeneity was tested with the Chi-square test and *I*^2^. The value of *I*^2^ < 25% was considered low statistical heterogeneity; *I*^2^ < 50%, moderate statistical heterogeneity; *I*^2^ < 75%, high statistical heterogeneity [15].

## Results

### Study selection

**Figure 1:**
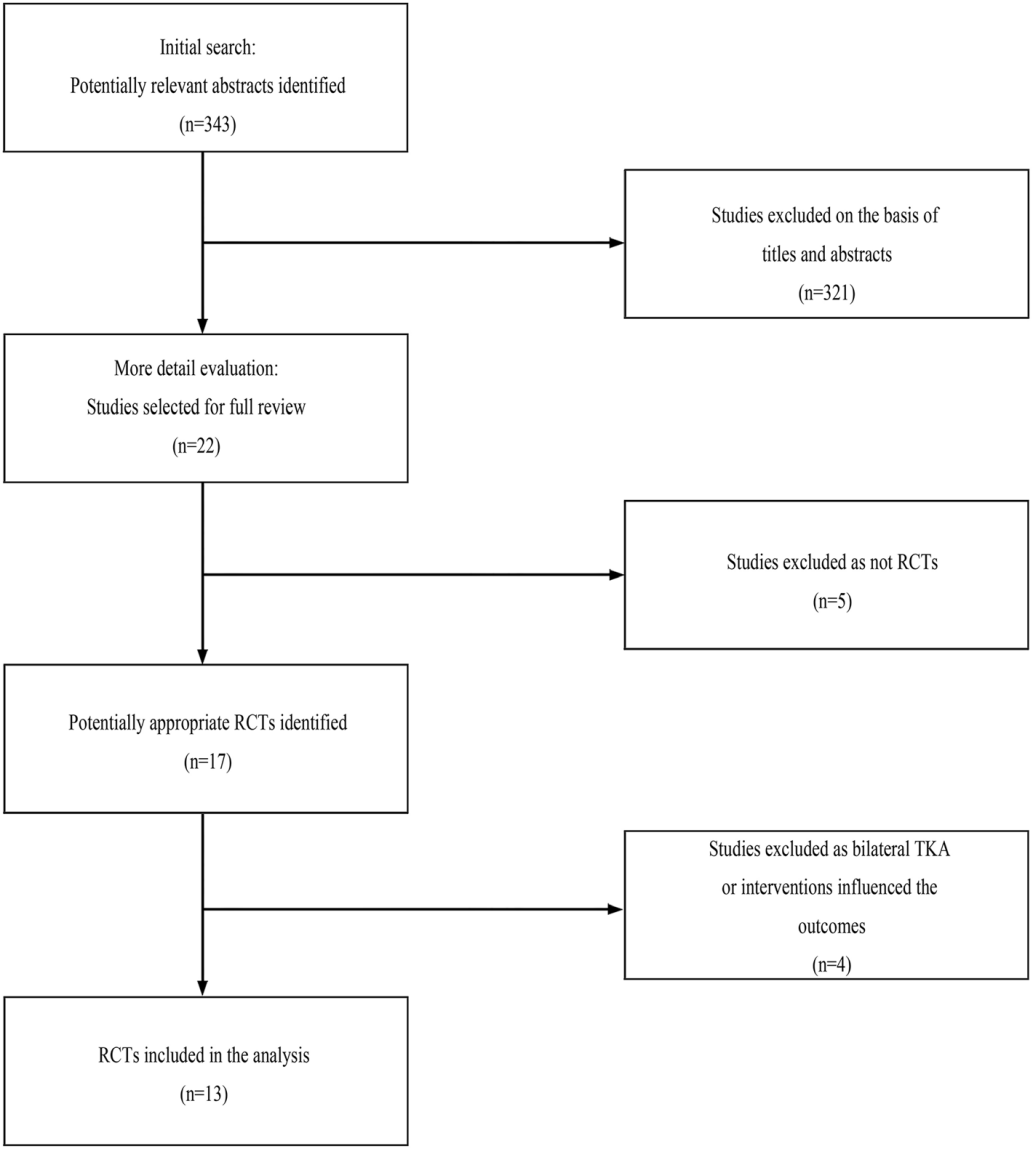
Flow chart summarizing the selection process of randomized control trials (RCTs).

A total of 343 articles were identified for the meta-analysis. Of these, 321 studies were excluded because the titles or abstracts did not meet the eligibility criteria. Then, 22 papers were retrieved in full text. Five literatures were not RCTs, and two trials included bilateral TKA during the same surgery [16,17]. What is more, two studies were excluded for some intervening measures with potential to influence the result [18,19]. For one, in Kiss et al.'s study, the non-tourniquet group used epinephrine-augmented hypotensive epidural anesthesia, and the tourniquet group patients received normotensive epidural anesthesia [18]. Epinephrine-augmented hypotensive epidural anesthesia could affect the blood loss [20], which might influence on other aspects of patients. For other, in Padala et al.'s study, the non-tourniquet group received 2.5 mg of adrenaline diluted in 500 ml of normal saline which was then infiltrated into the skin, subcutaneous tissues, and capsule before surgical incision. However, the tourniquet group did not receive it [19]. As a result, the aforementioned nine studies were excluded. Finally, 13 RCTs comparing the effects of using a tourniquet or not in TKA were included in the meta-analysis. A summary of the review process was presented (Figure 1). All of the papers were published in English. The dataset included 689 patients (689 knees). The study included 351 TKAs with a tourniquet compared with 338 TKAs without a tourniquet.

### Critical appraisal

The methodological quality of each included RCTs was assessed in accordance with the PEDro scale (Table 1). The results showed that 11 RCTs were of high quality and only 2 were of low quality. All trials used randomized method and six of them described the details of randomized method. Six studies used concealed allocation. Eight studies used the blinding method.

**Table 1:** PEDro critical appraisal scores

**Study**	**PEDro critical appraisal score**^**a**^											**Total**
	**1**	**2**	**3**	**4**	**5**	**6**	**7**	**8**	**9**	**10**	**11**	
Tai [21]	Y	Y	Y	Y	Y	N	Y	Y	Y	Y	Y	9
Ledin [22]	Y	Y	Y	Y	Y	N	N	Y	N	Y	Y	7
Zhang [23]	Y	Y	N	Y	N	N	N	Y	Y	Y	Y	6
Li [24]	Y	Y	N	Y	Y	N	N	Y	Y	Y	Y	7
Kageyama [25]	Y	Y	Y	Y	Y	N	Y	Y	Y	Y	Y	9
Matziolis [26]	Y	Y	N	Y	Y	N	N	Y	Y	Y	Y	8
Aglietti [27]	Y	Y	N	Y	N	N	N	Y	Y	Y	Y	6
Tetro [5]	Y	Y	Y	Y	Y	N	Y	Y	Y	Y	Y	9
Clarke [28]	Y	Y	Y	N	Y	N	N	Y	N	Y	Y	6
Wauke [29]	Y	Y	N	N	N	N	N	Y	Y	Y	Y	5
Vandenbussche [30]	Y	Y	Y	Y	Y	N	N	Y	Y	Y	Y	8
Wakankar [31]	Y	Y	N	Y	N	N	N	Y	Y	Y	Y	6
Abdel-Salam [32]	Y	Y	N	Y	N	N	N	Y	N	Y	Y	5

^a^PEDro criteria: (1) eligibility criteria, (2) random allocation, (3) concealed allocation, (4) baseline comparability, (5) participant blinding, (6) therapist blinding, (7) assessor blinding, (8) >85% follow-up, (9) intention-to-treat analysis, (10) between-groups statistical comparison for at least one key outcome, (11) point estimates and variability measures for at least one key outcome. *N* no, *PEDro* Physiotherapy Evidence Database scale, *Y* yes.

### Outcomes

**Figure 2:**
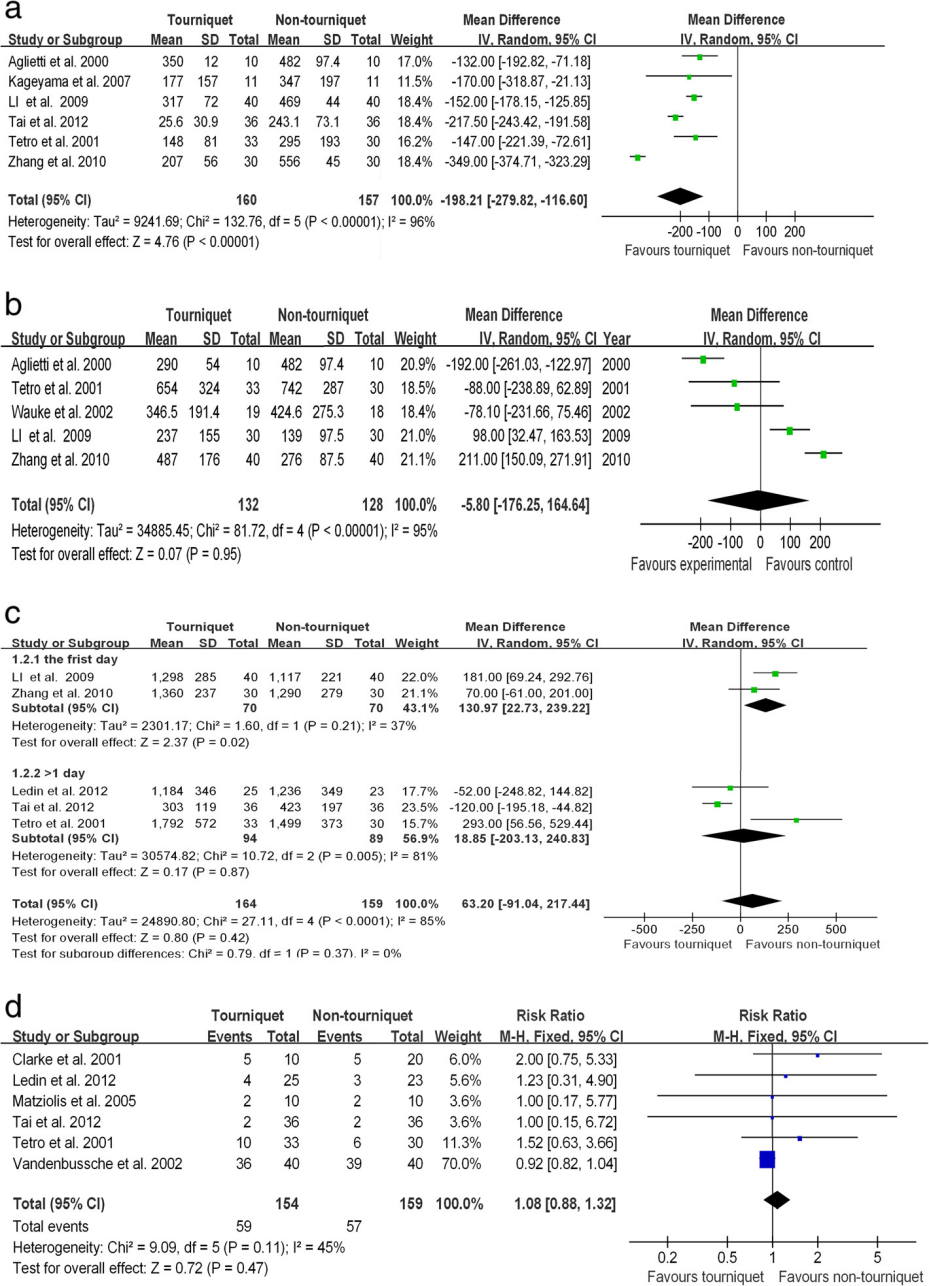
**Forest plot for blood loss. (a)** Intraoperative blood loss between TKA with a tourniquet and TKA without a tourniquet. **(b)** Postoperative visible blood loss between TKA with a tourniquet and TKA without a tourniquet. **(c)** Calculated blood loss between TKA with a tourniquet and TKA without a tourniquet. **(d)** Rate of transfusion between TKA with a tourniquet and TKA without a tourniquet. *CI* confidence interval, *IV* inverse variance.

The intraoperative blood loss was described in eight studies, but two of them were excluded because the intraoperative blood loss of one research was estimated by the anesthetists using their experience to estimate how much blood loss was in the soaked sponges and another research did not record blood loss in sponges [30]. The pooling data showed that using a tourniquet in TKA significantly decreased intraoperative blood loss by 198.21 ml (WMD, -198.21; 95% CI, -279.82 to -116.60; *P* < 0.01) (Figure 2a).

Five papers described the postoperative visible blood loss. They included soaked dressings blood and suction drains blood loss. The Forest plot of postoperative visible blood loss showed no significant difference between TKA with a tourniquet and without a tourniquet (WMD, -5.80; 95% CI, -176.25 to -164.64; *P* = 0.95) (Figure 2b).

Five studies reported calculated blood loss. Because high statistical heterogeneity existed among these studies (*P* < 0.01; *I*^2^ = 85%), a subgroup analysis based on the calculative timing of the calculated blood loss was done. Two trials calculated total blood loss from hemoglobin and hematocrit values at 24th postoperative hour [23,24]. However, the other three studies calculated it from the lowest hemoglobin and hematocrit levels on postoperative day 4 or 7 [5,21,22]. Therefore, the five studies were divided into these two subgroups. There were no significant difference between TKA with a tourniquet and TKA without a tourniquet (WMD, 63.20; 95% CI, -91.04 to 277.44; *P* = 0.80) (Figure 2c).

Seven studies mentioned the incidence of transfusion. Yet, one study was excluded for the reason that patients had preoperative autologous donation of blood [30]. The pooling result showed no statistical difference in ratio of transfusion between the two groups (RR, 1.27; 95% CI, 0.66 to 2.43; *P* = 0.47) (Figure 2d).

**Figure 3:**
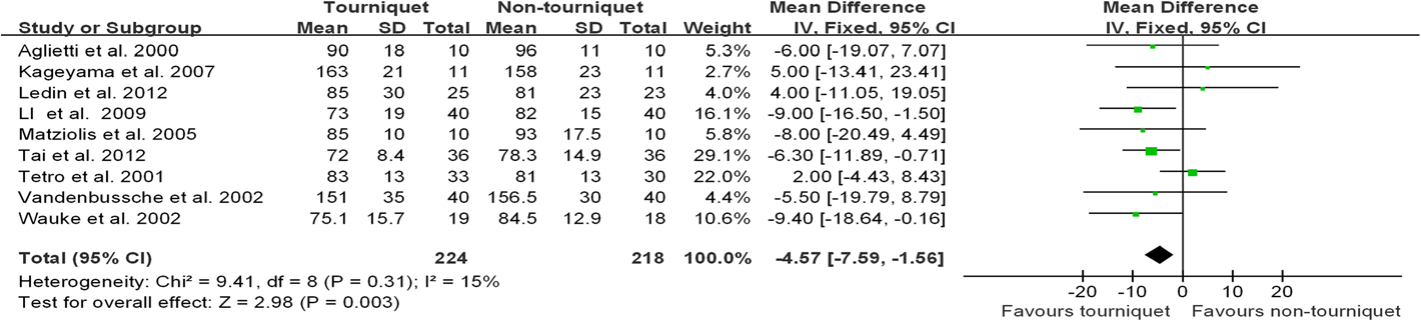
**Forest plot for operation time between TKA with a tourniquet and TKA without a tourniquet.** *CI* confidence interval, *IV* inverse variance.

Nine trials recorded the operation time. The operation time in tourniquet group was 4.57 min less than non-tourniquet group (WMD, -4.57; 95% CI, -7.59 to -1.56; *P* < 0.01) (Figure 3).

**Figure 4:**
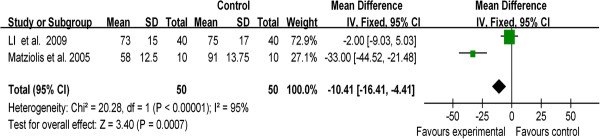
**Forest plot for postoperative knee ROM in early stage between TKA with/without a tourniquet.** *CI* confidence interval, *M-H* Mantel-Haenszel statistics.

Postoperative ROM in early stage (≤10 days after surgery) was reported in five studies. However, three studies did not have standard deviation [22,31,32]. Therefore, the meta-analysis was performed on the remaining two studies. The pooling result showed postoperative ROM in early stage of TKA with a tourniquet was 10.41° less than that of TKA without a tourniquet (WMD, -10.41; 95% CI, -16.41 to -4.41; *P* < 0.01) (Figure 4).

**Figure 5:**
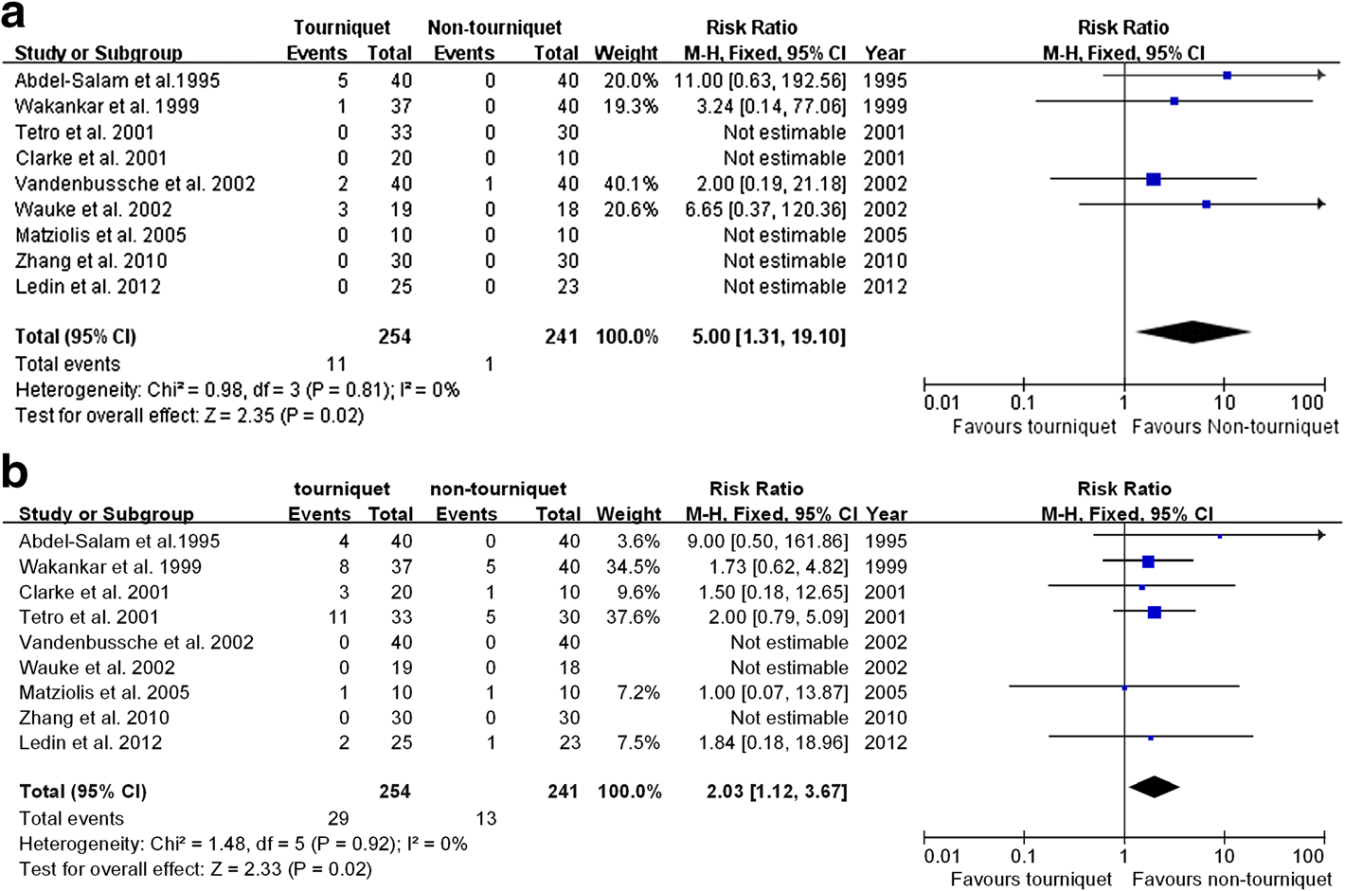
**Forest plot for thrombotic events and incidence of other complications between TKA with/without a tourniquet. (a)** Forest plot for thrombotic events between TKA with a tourniquet and TKA without a tourniquet. **(b)** Forest plot for the incidence of the other complications between TKA with a tourniquet and TKA without a tourniquet. *CI* confidence interval, *M-H* Mantel-Haenszel statistics.

Venous throboembolism (VTE) is the most common complication following TKA. Manifesting as DVT or PE, VTE is a leading cause of medical morbidity and mortality [33]. Therefore, for the current analysis, we divided the complications into two parts, namely, thrombotic events and non-thrombotic complications. DVT and PE were included in thrombotic events. Cases of thrombotic events were reported in nine studies. The pooling data stated that using a tourniquet in TKA significantly increased the risk of thrombosis compared to TKA without a tourniquet (RR, 5.00; 95% CI, 1.31 to 19.10; *P* = 0.02) (Figure 5a). The incidence of the non-thrombotic complications, (i.e*.*, infection, blister, hematoma, wound oozing, bruising, nerve palsy, reoperation, etc.) showed statistical difference between the two groups (RR, 2.03; 95% CI, 1.12 to 3.67; *P* = 0.02) (Figure 5b). Therefore, the result of the current meta-analysis indicated that TKA performed using a tourniquet could increase the incidence of postoperative complications.

## Discussion

The most significant finding of this study was that TKA with a tourniquet could increase postoperative complications. In addition, using a tourniquet in TKA could not reduce the total blood loss, although it could decrease the intraoperative blood loss. At the same time, postoperative ROM in tourniquet group was less than that in non-tourniquet group in the early stage, which indicated that the use of a tourniquet in TKA might hinder patients' early postoperative exercises.

Regarding the complications, the results of this meta-analysis confirm that there was a greater complication incidence in tourniquet-assisted procedures.

During the operation, thrombosis is a common and potentially fatal complication. Our result confirmed that there was a greater incidence when a tourniquet was used in TKA (RR = 5.00; *P* = 0.02). The finding of the current study was similar to the result disclosed by Parmet et al. that tourniquets used in patients were 5.33-fold greater risk of having a large emboli compared with TKA without a tourniquet [34]. There were also some strong evidences that tourniquet group could lead to a higher risk of thromboembolic events [31,32,35,36]. Our study indicated that using a tourniquet in TKA might increase the morbidity rate. The reasons are as follows: The formation of thrombi is associated with the triad of venous stasis, endothelial injury, and hypercoagulability, which is present in patients being managed with TKA [37]. A tourniquet can cause venous stasis, endothelial damage via direct trauma, and possible damage to calcified blood vessels. Zahavi et al. reported that ischemia from tourniquet use increases levels of plasma beta-thrombolobulin and plasma thromboxane-B2, thus increasing the risk of thrombosis in patients undergoing TKA [38]. In addition, Katsumata et al. found that during TKA, the use of a tourniquet might promote the local release of neutrophil elastase from the neutrophils together with reactive-oxygen derivatives, which can contribute to the development of DVT, PE, and tissue injury [39].

The complications were recorded in 13 RCTs. The pooling data showed that the tourniquet group had a greater risk of non-thrombotic complications compared with the non-tourniquet group (RR = 2.03; *P* = .02). Our finding was in accordance with the earlier published studies [40,41]. Olivecrona et al. reported the tourniquet time and cuff pressure were significantly associated with an increased risk of complications after TKA [8,42,43]. The use of a tourniquet in TKA was identified as a risk factor for the complications. The reasons are as follows: Firstly, the direct pressure of a tourniquet damages the nerves and local soft tissues [44]. Secondly, reactive hyperemia and increased fibrinolytic activity occur after tourniquet release increased the tissue pressure and local inflammation leading to tissue hypoxia and subsequently compromised wound healing [28,45]. Finally, the use of a tourniquet tethers the quadriceps mechanism and thus alters the intraoperative patellofemoral tracking. Then, this might affect the surgeon's judgment on soft tissue balancing and result in the unnecessary performance of a lateral release [46], which might have a detrimental effect on patellar viability [47] and could increase the incidence of hematomas requiring drainage and wound edge avascularity [48].

This study revealed that the use of a tourniquet in TKA was not associated with the total blood loss, which is consistent with a previous meta-analysis [2]. Although there is a contradictory meta-analysis about the effect of a tourniquet on total blood loss following TKA [40], some studies of this meta-analysis might had ignored the hidden blood loss, when they calculated the overall blood loss just by adding up the intraoperative blood loss and postoperative visible blood loss together. According to the result of our study, using a tourniquet in TKA decreased the intraoperative blood loss by 232.4 ml compared to TKA without a tourniquet. There was no significant difference in postoperative visible blood loss and overall blood loss. Therefore, it can be explicitly deduced that TKA with a tourniquet could increase postoperative hidden blood loss. The result of the current study is consistent with Tetro et al.'s [5] and Li et al.'s findings [24]. Sehat et al. reported that the total blood loss included the visible blood loss and the hidden blood loss, and what is more, the mean hidden loss is 50% of the total blood loss [49]. The mechanism of the hidden blood loss was generally accepted as the residual blood into the joint and extravasations into the tissue [50]. After the tourniquet was released, the reactive blood flow reaches its peak within 5 min [51] and postischemic reactive hyperemia reflects the body's attempt to cleanse the limb of the metabolic products of anoxia and according for approximately one half of the hidden blood loss [52]. Meanwhile, the increased fibrinolytic activity associated with tourniquet-induced ischemia promotes bleeding into the local tissues following the procedure [27,53,54].

Regarding transfusion, it was broadly related to the overall blood loss and there was no significant difference between the tourniquet group and non-tourniquet group following the total blood loss.

Therefore, although the use of a tourniquet reduced the intraoperative blood loss, it could not decrease the total blood loss and the incidence of transfusion effectively.

For operation time, it mainly depends on the surgical technique of the surgeons in the uncomplicated TKA. This meta-analysis found that the operation time was shorter in tourniquet group than that in the non-tourniquet group. The application of a tourniquet in TKA was believed to be effective for providing a relative bloodless field, which facilitate saving surgical time. In this current study, there were two RCTs that confirmed that non-tourniquet resulted in less operation time [2,55], whereas no significant difference was observed in other six RCTs [5,22,25-27,29,30]. However, the pooling result showed that TKA with a tourniquet could save the operation time for only 4.57 min compared to TKA without a tourniquet, which had no clinical significance.

For ROM, Forest plot showed that the postoperative ROM in tourniquet group was 10.41° less than that in non-tourniquet group in the early stage. The similar result recorded by Wakankar et al. showed 9.48° difference in favor of TKA without tourniquet over TKA with a tourniquet [31]. There were also several strong evidences from other RCTs that non-tourniquet group resulted in a better ROM [22,31,32]. Meanwhile, the result by Ledin et al. showed the ROM was less in tourniquet group than that in non-tourniquet group from 3 days to 2 years post-operatively [22], which indicated that TKA with a tourniquet might hamper patients' early postoperative exercises. The mechanism behind the reduce ROM is not very clear. The possible reasons are as follows: (1) Using a tourniquet could injure the nerve and the skeletal muscle, even causing rhabdomyolysis [56]. The delay in the nerve conduction and the changes by electromyography of the extensor apparatus have been recorded in tourniquet group [7,57]. The result by Liu et al.'s trial showed that non-tourniquet group could support more energy in their quadriceps muscle than the tourniquet group [7]. (2) The direct damage of the tourniquet and reperfusion injury might increase pain that would reduce the patient's ability to perform postoperative training [22]. (3) TKA with a tourniquet could increase postoperative additional hidden blood loss compared to TKA without a tourniquet. Then, the additional hidden blood loss escapes into the joint space and the soft tissue that would result in limb swelling [50]. Additional swelling might produce an increased weight in the affected limb sufficient to require more muscle force for conducting straight-leg raising activities. Meanwhile, it would also increase the tension of soft tissues, which lead to oxygen tension decrease [55].

There is less blood in the operating field with a tourniquet, which theoretically is to achieve better cementation [40]. However, it is unknown at present if TKA without a tourniquet could potentially lead to increased component loosening over time [58]. Furthermore, the good cement-bone interface shear strength during hip arthroplasty is obtained [59]. The study by Ledin et al. showed that there was no difference between the tourniquet group and the non-tourniquet group in migration, which was measured by radiostereometric analysis in 2 years postoperation [22]. The current meta-analysis had several strengths. First, all 13 included studies were RCTs, about 85% of which were of high methodological quality; second, the total blood loss was calculated by Gross formula [60], which is close to the actual situation; third, there were many controversies in the effect of simultaneous and staged TKA [61]. Therefore, we excluded the studies of bilateral TKA, which made the results more precise.

The limitations of this analysis are the following: Firstly, the study was limited to the literatures published in English. Selection bias in language must have existed. Secondly, there were some biases in these studies that might come from the measurement of the total blood loss of our study. The reason may be that the perioperative blood loss in TKA patients was associated with not only the application of tourniquet but also the other perioperative management, such as the drain [62], anticoagulant therapy [63], and the timing of lower limb tourniquet application [17,64] (Table 2).

**Table 2:** Study characteristic

**Study**	**Sample size**		**Total size**	**Gender M/F ratio**		**Mean age (years)**		**BMI (kg/m**^**2**^**)**		**Drain**	**Tourniquet duration**	**Anticoagulation**
	**T**	**NT**		**T**	**NT**	**T**	**NT**	**T**	**NT**			
Tai [21]	36	36	72	9/27	8/28	72.1	71.5	28.6	27.9	N	Part	None
Ledin [22]	25	23	48	10/15	9/14	70.0	71.0	29.0	28.0	Y	Overall	Heparin
Zhang [23]	30	30	60	8/22	11/19	72.0	71.0	25.0	26.0	Y	Overall	Heparin
Li [24]	40	40	80	11:29	3/13	71.0	70.0	27.3	26.8	N	Part	Heparin
Kageyama [25]	11	11	22	2/9	2/9	73	76.0	NS	NS	NS	NS	NS
Matziolis [26]	10	10	20	2/8	3/7	72.4	76.6	28.3	29.5	NS	NS	NS
Aglietti [27]	10	10	20	3/7	4/6	70.0	68.0	27.9	27.3	Y	Part	NS
Tetro [5]	33	30	63	15/18	11/19	69.8	69.8	NS	NS	Y	Part	Coumarins
Clarke [28]	20	10	30	NS	NS	NS	NS	NS	NS	Y	NS	NS
Wauke [29]	19	18	37	0/19	0/18	63.2	61.4	NS	NS	NS	NS	Heparin
Vandenbussche [30]	40	40	80	9/31	16/24	72.5	68.5	NS	NS	Y	Overall	Heparin
Wakankar [31]	37	40	77	11/26	14/26	72.5	71.8	NS	NS	Y	NS	Warfarin
Abdel-Salam [32]	40	40	80	17/23	15/25	72.0	74.0	NS	NS	Y	Overall	Heparin

*T* TKA with a tourniquet group, *NT* TKA without a tourniquet group, *N* no drainage system was used in TKA, *Y* drainage system was used in TKA, *Part* tourniquet was not inflated during the entire operation, *Overall* tourniquet was inflated during the entire operation, *NS* not stated.

## Conclusions

This meta-analysis demonstrates that non-tourniquet use in TKA has better clinical outcomes with regard to the incidence of complications and ROM in early postoperative period. There was no significant difference between the two groups in the actual blood loss. Therefore, considering the effectiveness and safety of the use of a tourniquet in TKA, the surgeons should use it prudently.

## Competing interests

The authors declare that they have no competing interests.

## Authors' contributions

WZ carried out the entire procedure including the literature search, data extraction, performed the statistical analysis, drafted the manuscript, revised submitted the manuscript. LC conceived of the study, coordinated and participated in the entire process of drafting and revised the manuscript. NL contributed to statistical analysis and revision the manuscript. SC contributed to the literature search, data extraction and the statistical analysis. YT and MAA contributed to the revisions of the manuscript. All authors have contributed significantly. All authors read and approved the final manuscript.
